# Supplementary material for: Development of deep learning algorithms for predicting blastocyst formation and quality by time-lapse monitoring
Source: Commun Biol. 2021 Mar 26;4:415. doi: 10.1038/s42003-021-01937-1 (PMC7998018; doi:10.1038/s42003-021-01937-1)
Supplement: Supplementary file 2 — Supplementary Information [file 42003_2021_1937_MOESM2_ESM.pdf]

Development of Deep Learning Algorithms for Predicting Blastocyst Formation and Quality by Time-lapse Monitoring

Qiuyue Liao[#1], Qi Zhang[#2], Xue Feng[#1], Haibo Huang[#2], Haohao Xu[#2], Baoyuan Tian[2], Jihao Liu[2], Qihui Yu[2], Na Guo[1], Qun Liu[1], Bo Huang[1], Ding Ma[1], Jihui Ai*[1], Shugong Xu*[2], Kezhen Li *[1]

[1]Department of Gynecology and Obstetrics, Tongji Hospital, Tongji Medical College, Huazhong University of Science and Technology, Wuhan, Hubei 430030, China

[2]Shanghai Institute for Advanced Communication and Data Science, Shanghai University, Shanghai 200444, China

Kezhen Li, Email:tjkeke@126.com

Shugong Xu, Email: shugong@shu.edu.cn

Jihui Ai, Email: jihuiai@tjh.tjmu.edu.cn

Contributor Information.

*These authors jointly supervised this work.

[#]These authors contributed equally.

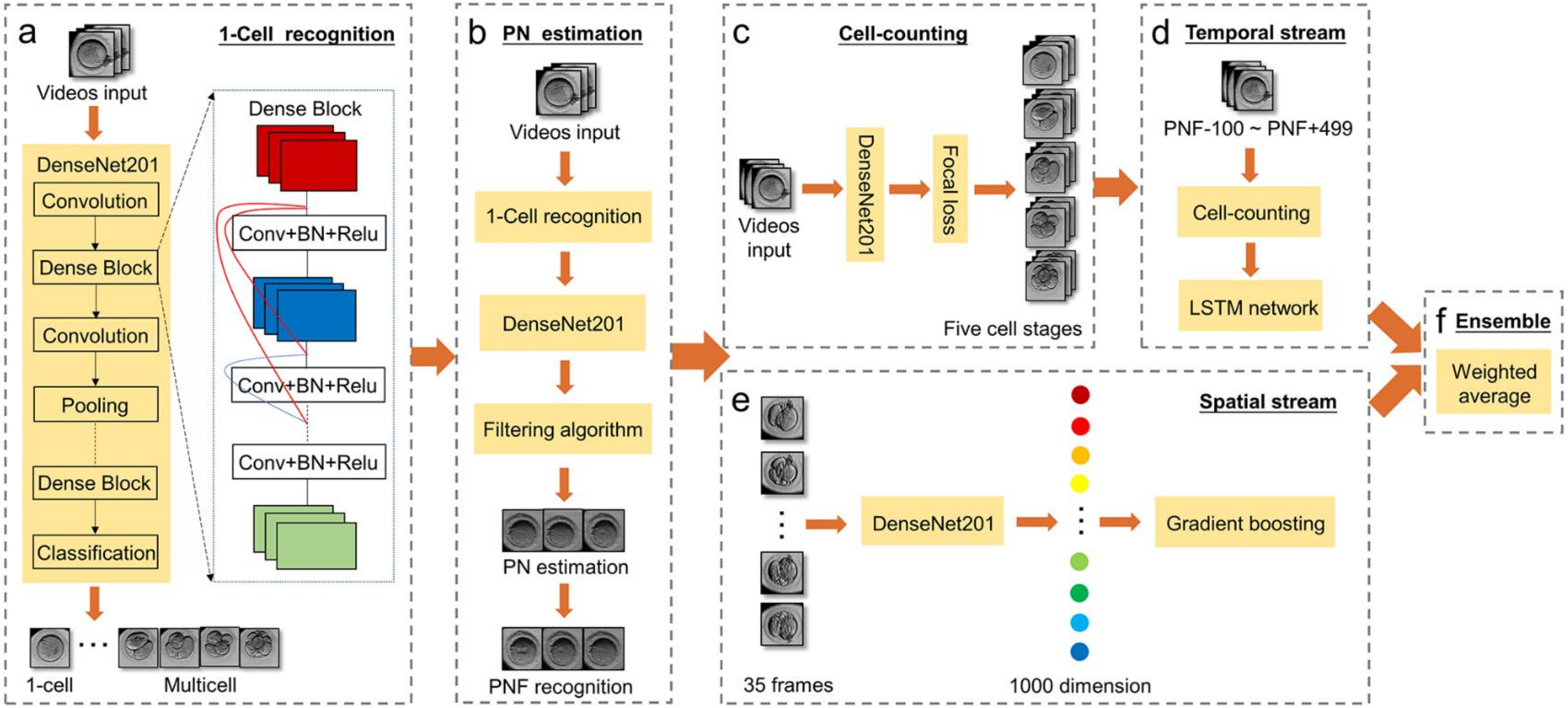

Supplementary Figure 1. Overall framework of prediction model. The prediction model is trained by 5 steps. **a**, A 1-cell recognition algorithm was developed using DenseNet201. **b**, A PN estimation algorithm was developed using DenseNet201 network based on the 1-cell stage frames. **c**, A cell-counting model was built using the DenseNet201 network and focal loss. **d**, A cell-counting model combined with LSTM network were used to train a temporal stream model. **e**, A spatial stream model was established using the DenseNet201 network and gradient boosting classifier. **f**, The spatial stream model and temporal stream model were combined as a final prediction model by weighted average.

Supplementary table 1. The outcomes of single or multiple cells recognition in 264 videos.

Stages labeled by embryologists	Stages recognized by AI		Total frames	Sensitivity	Specificity	PPV	NPV	Accuracy
	1-cell	Muti-cell						
1-cell	75232	839	76071	98.9%	99.6%	99.3%	99.4%	99.4%
Muti-cell	524	140747	141271					

Note: PPV: Positive predictive value, NPV: Negative predictive value.

Supplementary table 2. The performance of PN estimation model to recognize PN in 264 videos.

Frames labeled by embryologists	Frames recognized by AI		Total frames	Sensitivity	Specificity	PPV	NPV	Accuracy
	Presence	Absence						
Presence	46130	2514	48644	94.8%	90.9%	94.9%	90.8%	93.4%
Absence	2494	24933	27427					

Note: PPV: Positive predictive value, NPV: Negative predictive value.

Supplementary table 3. The performance of cell-counting model to recognize cell stages in 114 videos.

Frames labeled by embryologists	Number of recognized frames by AI					Total frames	Sensitivity	PPV
	1-cell	2-cell	3-cell	4-cell	≥ 5-cell			
1-cell	33014	272	147	284	249	33966	97.2%	99.5%
2-cell	39	11044	1094	287	37	12501	88.3%	96.1%
3-cell	0	169	4241	304	14	4728	89.7%	75.0%
4-cell	1	7	166	13478	874	14526	92.8%	92.3%
≥ 5-cell	111	2	9	243	14214	14579	97.5%	92.4%
Overall accuracy	94.6%							

Note: PPV: Positive predictive value.

Supplementary table 4. The performances of AI models for predicting blastocyst formation (training dataset n=8346, validation dataset n=2086)

	Accuracy	Sensitivity	Specificity	PPV	NPV
Temporal stream network	76.9%	84.7%	64.7%	78.7%	73.3%
Spatial stream network	70.0%	70.4%	69.5%	81.5%	68.3%
Ensemble result	78.2%	85.9%	66.3%	79.7%	75.3%

Note: PPV: Positive predictive value, NPV: Negative predictive value.

Supplementary table 5. The prediction results for blastocyst formation from the STEM and the four embryologists.

	STEM		Embryologist I		Embryologist II		Embryologist III		Embryologist IV	
Actual outcome	B	NB	B	NB	B	NB	B	NB	B	N
B n=1265	1087	178	942	323	855	410	935	330	834	431
NB n=821	277	544	348	473	331	490	390	431	301	520
Sensitivity	85.9%		74.5%		67.6%		73.9%		65.9%	
Specificity	66.3%		57.6%		59.7%		52.5%		63.3%	
PPV	79.7%		73.0%		72.1%		70.6%		73.5%	
NPV	75.3%		59.4%		54.4%		56.6%		54.7%	
Accuracy	78.2%		67.8%		64.5%		65.5%		64.9%	

Note: B: blastocyst, NB: Nonblastocyst. PPV: Positive predictive value, NPV: Negative predictive value.

Supplementary table 6. The performances of AI models for predicting usable blastocyst formation (training dataset n=8346, validation dataset n=2086)

	Accuracy	Sensitivity	Specificity	PPV	NPV
Temporal stream network	71.8%	74.1%	70.6%	57.6%	83.5%
Spatial stream network	68.6%	77.3%	64.0%	53.6%	83.9%
Ensemble result	71.9%	75.5%	70.0%	57.5%	84.1%

Note: PPV: Positive predictive value, NPV: Negative predictive value.
