# Supplementary material for: Development of deep learning algorithms for predicting blastocyst formation and quality by time-lapse monitoring
Source: Commun Biol. 2021 Mar 26;4:415. doi: 10.1038/s42003-021-01937-1 (PMC7998018; doi:10.1038/s42003-021-01937-1)
Supplement: Supplementary file 3 — Description of Additional Supplementary Files [file 42003_2021_1937_MOESM3_ESM.pdf]

Description of Additional Supplementary Files

File name: Supplementary Data 1

Description: *Results for models.*

Sheet 1, frames of PNF labeled by embryologists and predicted by PNF estimation algorithm; Sheet 2, outcomes of embryos predicted by STEM and human embryologists. “1” represents blastocyst and “0” represents nonblastocyst; Sheet 3, outcomes of embryos predicted by STEM+ and outcomes of implantation of usable blastocysts. “1” represents usable blastocyst and “0” represents unusable blastocyst.
