# Supplementary material for: Development of deep learning algorithms for predicting blastocyst formation and quality by time-lapse monitoring
Source: Commun Biol. 2021 Mar 26;4:415. doi: 10.1038/s42003-021-01937-1 (PMC7998018; doi:10.1038/s42003-021-01937-1)
Supplement: Supplementary file 5 — Reporting Summary [file 42003_2021_1937_MOESM5_ESM.pdf]

nature research

Corresponding author(s): Kezhen Li, Shugong Xu, Jihui Ai

Last updated by author(s): Feb 13, 2021

Reporting Summary

Nature Research wishes to improve the reproducibility of the work that we publish. This form provides structure for consistency and transparency in reporting. For further information on Nature Research policies, see our Editorial Policies and the Editorial Policy Checklist.

Statistics

For all statistical analyses, confirm that the following items are present in the figure legend, table legend, main text, or Methods section.

n/a	Confirmed	
☐	☒	The exact sample size (n) for each experimental group/condition, given as a discrete number and unit of measurement
☐	☒	A statement on whether measurements were taken from distinct samples or whether the same sample was measured repeatedly
☐	☒	The statistical test(s) used AND whether they are one- or two-sided<br>*Only common tests should be described solely by name; describe more complex techniques in the Methods section.*
☒	☐	A description of all covariates tested
☒	☐	A description of any assumptions or corrections, such as tests of normality and adjustment for multiple comparisons
☐	☒	A full description of the statistical parameters including central tendency (e.g. means) or other basic estimates (e.g. regression coefficient) AND variation (e.g. standard deviation) or associated estimates of uncertainty (e.g. confidence intervals)
☒	☐	For null hypothesis testing, the test statistic (e.g. F, t, r) with confidence intervals, effect sizes, degrees of freedom and P value noted<br>*Give P values as exact values whenever suitable.*
☒	☐	For Bayesian analysis, information on the choice of priors and Markov chain Monte Carlo settings
☐	☒	For hierarchical and complex designs, identification of the appropriate level for tests and full reporting of outcomes
☒	☐	Estimates of effect sizes (e.g. Cohen's d, Pearson's r), indicating how they were calculated

Our web collection on statistics for biologists contains articles on many of the points above.

Software and code

Policy information about availability of computer code

Data collection	All the data were collected by TLM system (Primo Vision; Vitrolife) .
Data analysis	We used python as program language. The frameworks were Keras and Scikit-learn, and the optimizer was Adam.

For manuscripts utilizing custom algorithms or software that are central to the research but not yet described in published literature, software must be made available to editors and reviewers. We strongly encourage code deposition in a community repository (e.g. GitHub). See the Nature Research guidelines for submitting code & software for further information.

Data

Policy information about availability of data

All manuscripts must include a data availability statement. This statement should provide the following information, where applicable:

- Accession codes, unique identifiers, or web links for publicly available datasets
- A list of figures that have associated raw data
- A description of any restrictions on data availability

Provide your data availability statement here.

Field-specific reporting

Please select the one below that is the best fit for your research. If you are not sure, read the appropriate sections before making your selection.

☒ Life sciences ☐ Behavioural & social sciences ☐ Ecological, evolutionary & environmental sciences

For a reference copy of the document with all sections, see nature.com/documents/nr-reporting-summary-flat.pdf

Life sciences study design

All studies must disclose on these points even when the disclosure is negative.

Sample size	This retrospective cohort study collected TLM videos and outcomes of embryos that were fertilized and cultured at Tongji hospital from February 2014 to December 2017. All couples who had embryos placed in TLM incubators and further cultured to D5/6 were included regardless of the parents' age, infertility reason, ovarian stimulation protocol and fertilization procedure. A total of 26113 embryos from 2594 IVF/ICSI cycles were included.
Data exclusions	The D3 transferred/cryopreserved/discarded embryos were eliminated, and the videos that embryos were misplaced, covered, or interrupted were discarded, finally 12912 videos were retained.
Replication	All videos used for modeling were divided into two datasets, the training dataset and validation dataset. The replicability was tested in the validation dataset.
Randomization	The data used for video preparation algorithms and cell-counting algorithm were randomly selected by computer.
Blinding	The investigators did not know the clinical information and the embryo outcomes during data collection and data analysis.

Reporting for specific materials, systems and methods

We require information from authors about some types of materials, experimental systems and methods used in many studies. Here, indicate whether each material, system or method listed is relevant to your study. If you are not sure if a list item applies to your research, read the appropriate section before selecting a response.

Materials & experimental systems

n/a	Involved in the study	
☒	☐	Antibodies
☒	☐	Eukaryotic cell lines
☒	☐	Palaeontology and archaeology
☒	☐	Animals and other organisms
☐	☒	Human research participants
☒	☐	Clinical data
☒	☐	Dual use research of concern

Methods

n/a	Involved in the study	
☒	☐	ChIP-seq
☒	☐	Flow cytometry
☒	☐	MRI-based neuroimaging

Human research participants

Policy information about studies involving human research participants

Population characteristics	This retrospective cohort study collected TLM videos and outcomes of embryos that were fertilized and cultured at Tongji hospital from February 2014 to December 2017. All couples who had embryos placed in TLM incubators and further cultured to D5/6 were included regardless of the parents' age, infertility reason, ovarian stimulation protocol and fertilization procedure.
Recruitment	A total of 26113 embryos from 2594 IVF/ICSI cycles were included. The D3 transferred/cryopreserved/discarded embryos were eliminated, and the videos that embryos were misplaced, covered, or interrupted were discarded, finally 12912 videos were retained.
Ethics oversight	This study was approved by the Ethical Committee of Tongji Hospital, Tongji Medical College, Huazhong University of Science and Technology (TJ-IRB20190317).

Note that full information on the approval of the study protocol must also be provided in the manuscript.
